# Supplementary material for: Search for a genetic cause of variably protease-sensitive prionopathy
Source: medRxiv. 2025 Mar 17:2024.12.12.24318867. Originally published 2024 Dec 14. Preprint. [Version 2] doi: 10.1101/2024.12.12.24318867 (PMC11661330; doi:10.1101/2024.12.12.24318867)
Supplement: Supplement 2 [file media-2.pdf]

SUPPLEMENT

Search for a genetic cause of variably protease-sensitive prionopathy

Lian et al 2025

Supplementary Figures: Surveillance Western blots of VPSPr brain material. Per a reviewer's request, we are providing images of Western blots performed for surveillance purposes at the National Prion Disease Pathology Surveillance Center on N=10 of the VPSPr cases described in this manuscript. Diagnosis of these cases was made through a combination of Western blot, histology, and immunohistochemistry. Each blot represents 1 VPSPr case. Key: +: proteinase K-treated; -: not proteinase K-treated; F: frontal cortex (from VPSPr case); C: cerebellum (from VPSPr case); O: occipital cortex (from VPSPr case); Cx1: cortex 1 (from VPSPr case); Cx2: cortex 2 (from VPSPr Case); N: negative control (non-prion brain); T1: sCJD type 1 positive control; T2: sCJD Type 2 positive control; PTA: indicates phosphotungstic acid precipitation of PrP^{Sc} was used. All blots used the 1E4 primary antibody as indicated at upper left. The volume of sample loaded is indicated at lower left. VPSPr cases generally have partially protease-resistant PrP^{Sc} observed (in the "+" condition) for at least some brain regions, but usually with higher amounts of sample loaded than are required to observe PrP^{Sc} in sCJD positive controls.

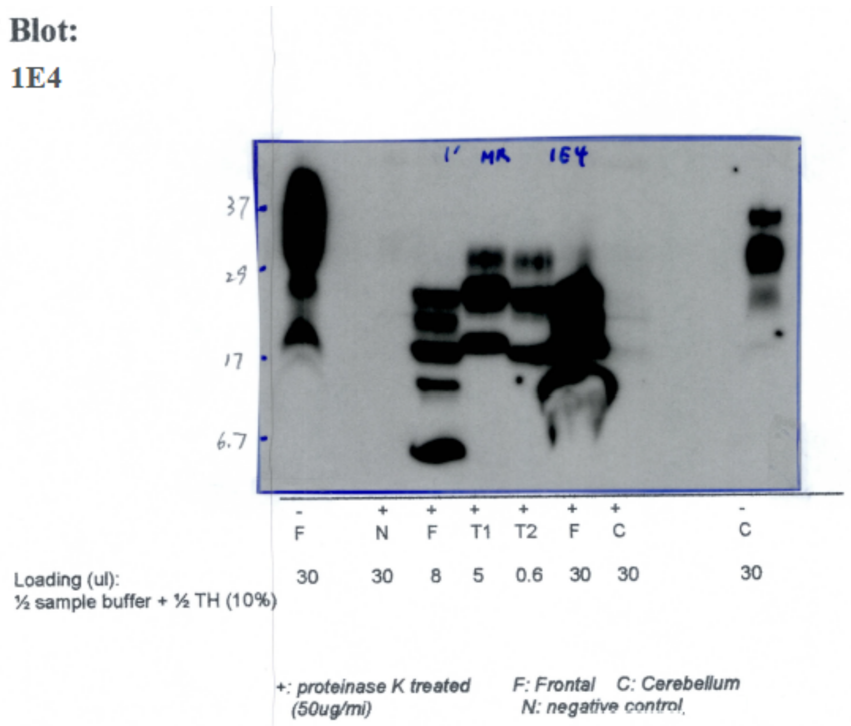

Blot:
1E4

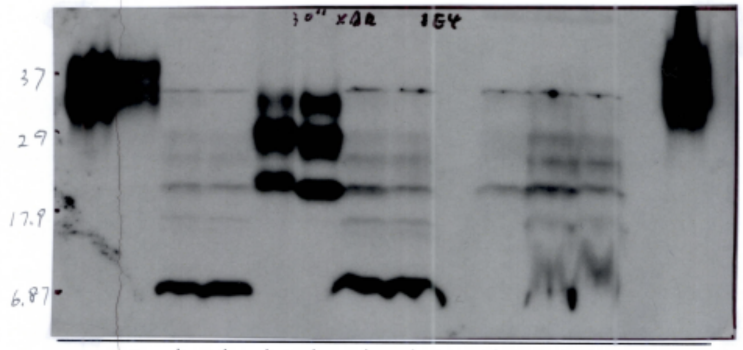

	-	-	+	+	+	+	+	+	+	+	+	-
	F	O	F	O	T1	T2	F	O	C	F	O	C
Loading (ul): ½ sample buffer + ½ TH (10%)	8	8	8	8	4	0.4	15	15	30	30	30	30

+: proteinase K treated (50ug/mi)   *F: Frontal*   *O: Occipital*   *C: Cerebellum*

Blot:
1E4

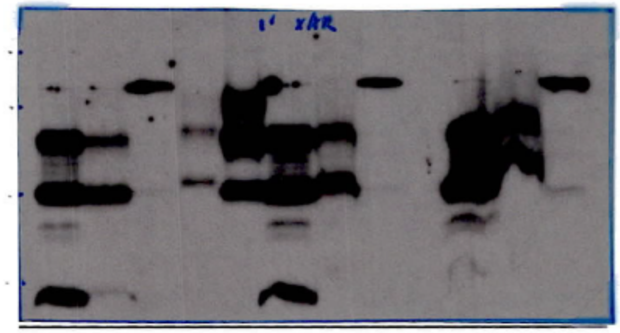

	+	+	+	+	+	+	+	+	+	+	+
	F	O	C	T1	T2	F	O	C	F	O	C
**Loading (ul): ½ sample buffer + ½ TH (10%)**	8	8	8	0.5	0.3	15	15	15	30	30	30

+: proteinase K treated (50ug/mi)   *F: Frontal*   *O: Occipital*   *C: Cerebellum*
N: negative control,

Blot:

1E4

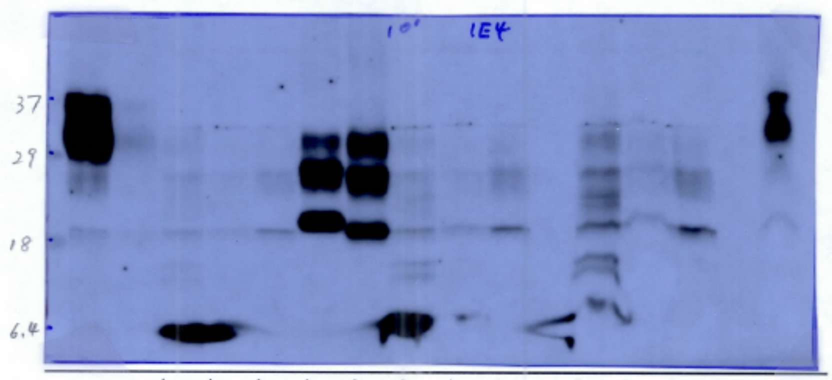

	-	-	+	+	+	+	+	+	+	+	+	+	+	-
	F	O	F	O	C	T1	T2	F	O	C	F	O	C	C
Loading (ul):<br>½ sample buffer + ½ TH (10%)	8	8	8	8	8	4.5	1.2	15	15	15	30	30	30	30

+: proteinase K treated (50ug/ml) *F: Frontal* *O: Occipital* *C: Cerebellum*

Blot:
1E4

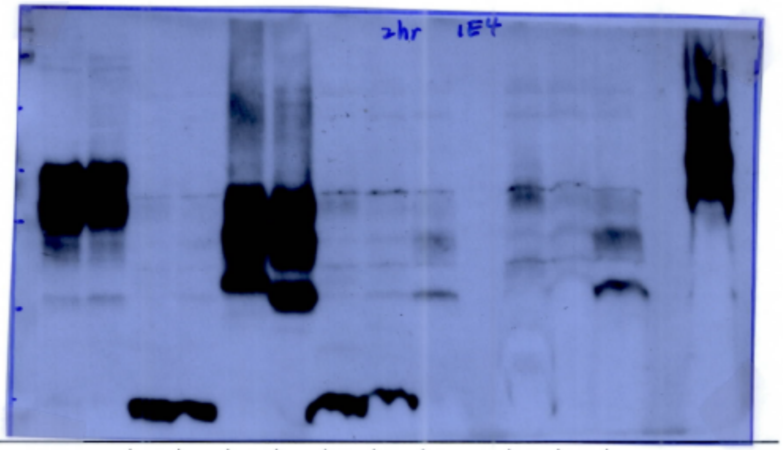

	-	-	+	+	+	+	+	+	+	+	+	+	-
	F	O	F	O	T1	T2	F	O	C	F	O	C	C
Loading (ul):<br>½ sample buffer + ½ TH (10%)	8	8	8	8	12	0.7	15	15	15	30	30	30	30

+: proteinase K treated (100ug/ml) *F: Frontal* *O: Occipital* *C: Cerebellum*

Blot:
1E4

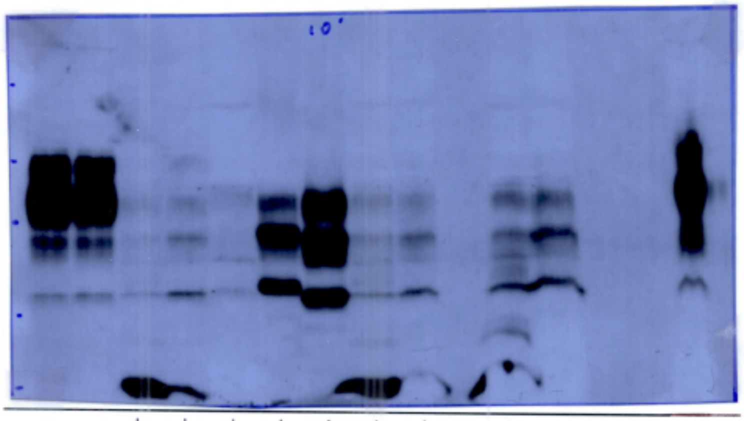

	-	-	+	+	+	+	+	+	+	+	+	-
	F	O	F	O	C	T1	T2	F	O	F	O	C
Loading (ul):<br>½ sample buffer + ½ TH (10%)	8	8	8	8	30	4.5	1.2	15	15	30	30	30

+: proteinase K treated (50ug/ml) *F: Frontal* *O: Occipital* *C: Cerebellum*

Blot:
1E4 PTA

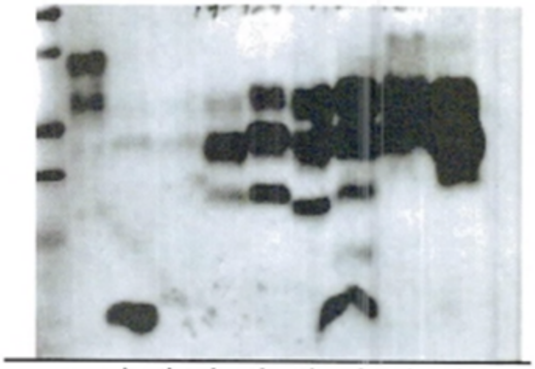

	-	+	+	+	+	+	+	+	+
	F	F	O	C	T1	T2	F	O	C
Loading (ul):	10	15	15	15	3.5	0.4	30	30	30

+: proteinase K treated   *F: Frontal* *O: Occipital* *C: Cerebellum*

Blot:
1E4

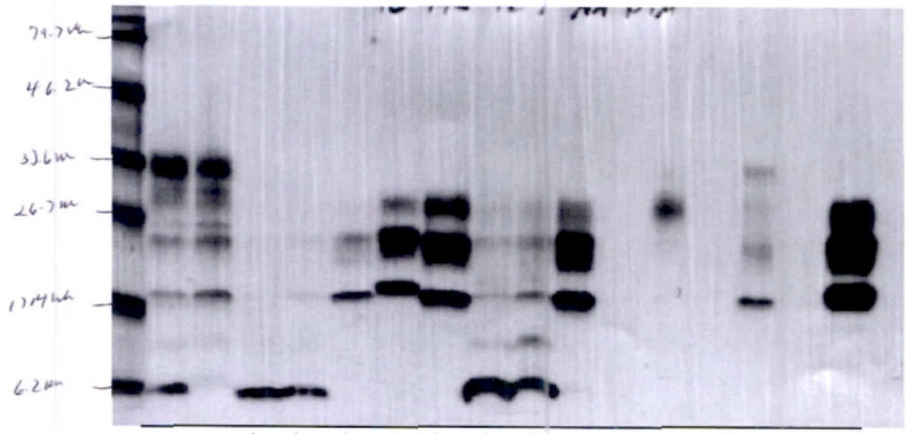

	-	-	+	+	+	+	+	+	+	+	+	-	+
	F	O	F	O	C	T1	T2	F	O	C	Neg	C	Pos
Loading (ul): ½ sample buffer + ½ TH (10%)	15	15	6	6	6	8	1	30	30	30	30	15	6

+: proteinase K treated   *F: Frontal*   *O: Occipital* *C: Cerebellum*

Blot:
1E4

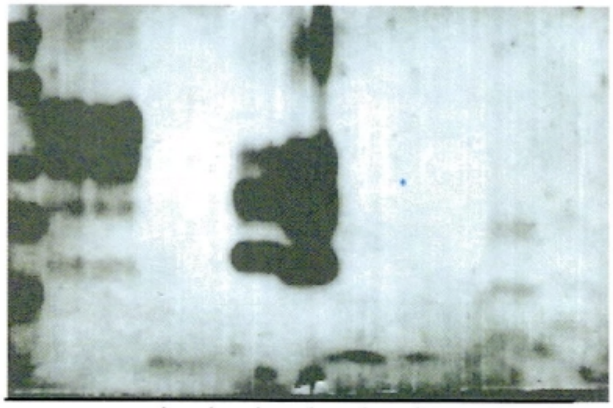

	-	-	+	+	+	+	+	+	+	+
	Cx1	Cx2	Cx1	Cx2	T1	T2	Cx1	Cx2	Cx1	Cx2
Loading (ul): ½ sample buffer + ½ TH (10%)	8	8	2	2	2	0.7	8	8	30	30

+: proteinase K treated *Cx1: Cortex1* *Cx2: Cortex2*

Blot:
1E4 PTA

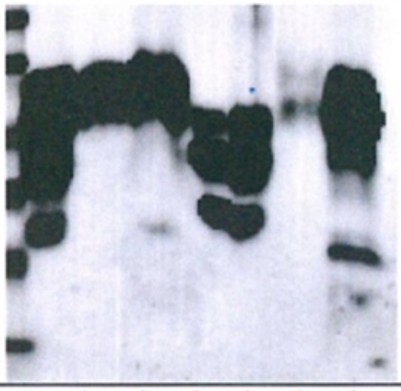

	-	+	+	+	+	+	+
	O	C	O	T1	T2	NC	F
Loading (ul):	1	30	30	1	0.2	30	25

+: proteinase K treated *F: Frontal O: Occipital C: Cerebellum*
NC: Negative control
